# “Eat and you will be eaten”: a qualitative study exploring costs and benefits of age-disparate sexual relationships in Tanzania and Uganda: implications for girls’ sexual and reproductive health interventions

**DOI:** 10.1186/s12978-018-0650-0

**Published:** 2018-12-13

**Authors:** Joyce Wamoyi, Ana Maria Buller, Daniel Nyato, Nambusi Kyegombe, Rebecca Meiksin, Lori Heise

**Affiliations:** 1 Department of Sexual and Reproductive Health, National Institute for Medical Research, PO Box 1462, Mwanza, Tanzania; 2 Department of Public Health, Environments and Society, London School of Hygiene and Tropical Medicine, 15-17 Tavistock Place, London, WC1H 9SH UK; 3 Department of Global Health and Development, London School of Hygiene and Tropical Medicine, 15-17 Tavistock Place, London, WC1H 9SH UK; 4 Johns Hopkins Bloomberg School of Public Health and Johns Hopkins School of Nursing, Department of Population, Family and Reproductive Health, Johns Hopkins Bloomberg School of Public Health, 615 N Wolfe Street, Baltimore, MD 21205 USA

**Keywords:** Transactional sex, Age-disparate sex, Adolescents, SRH, Young women, Interventions, Tanzania, Uganda

## Abstract

**Background:**

Age-disparate sex is associated with increased HIV risk among adolescent girls and young women (AGYW) in sub-Saharan Africa. However, little has been done to understand the dynamics of such relationships from the perspectives of either AGYW or older men, and the communities in which these relationships are embedded. This article explores the motivations and perceived benefits of such relationships for AGYW and older men, plus the social and sexual and reproductive health (SRH) consequences.

**Methods:**

This study held 37 participatory focus group discussions and 87 in-depth interviews with young people aged 14–24 and adult community members aged 25–49 in rural and urban Tanzania and Uganda. Participants were sampled using purposive and snowball techniques. Thematic analysis was conducted with the aid of NVIVO 10 software.

**Results:**

Motivations, perceived benefits and costs for AGYW centred around four main themes: financial motivations, emotional support, meeting social expectations and reflections on sexual health. Specifically, AGYW noted that older partners gave gifts/money of higher value compared with younger men. Men’s perceived benefits and costs revolved around the need to satisfy their sexual desire, the perception that AGYW were capable of engaging in new and creative sexual styles and their desire for prestige among male peers. Both AGYW and men recognised the social and SRH consequences as: risk of violence, social stigma, risk of unplanned pregnancy and risk of sexually transmitted infections including HIV.

**Conclusion:**

Interventions need to acknowledge the perceived benefits of age-disparate sexual relationships for AGYW and older men and engage them in critical reflection on the medium- to longer-term consequences versus the shorter-term satisfaction of needs, desires and aspirations, as a way to navigate the constrained opportunities they face given existing structural limitations. Interventions should also tackle the structural constraints AGYW face by helping them access resources, become empowered and challenge the expectation of having to depend financially on men. Interventions with men should unpack the assumption that men are naturally hypersexual. The role of peers for both girls and men should be acknowledged, and a shift from individual targeted interventions to changing norms at the community level should be considered.

## Plain English summary

Age-disparate sex is heterosexual intercourse with a non-marital partner ten plus years older. In sub-Saharan Africa, these relationships are characterised by romantic/sexual involvement between adolescent girls and younger women (AGYW) and older men and may involve transactional sex. These relationships have been found to increase AGYW’s risk of HIV and other sexual and reproductive health (SRH) problems.

This study sought the views of both groups on the motivations and perceived benefits of engaging in such relationships and on the social and SRH consequences.

Qualitative data collection included 37 participatory focus group discussions and 87 in-depth interviews with young people (female and male) aged 14–24 and adult men and women aged 25–49 in rural and urban Tanzania and Uganda.

AGYW identified the primary reasons for sex with older men as: financial benefit, emotional support and meeting social expectations. Adult men identified: pleasure; desire to “test new flavours”; prestige; and the belief that AGYW were HIV negative. The social and SRH consequences were understood to be: risk of coercion and violence, stigma, unplanned pregnancy and risk of sexually transmitted infections including HIV.

Interventions need to engage AGYW and older men in a critical reflection process on the consequences of engaging in age-disparate relationships, especially those that involve transactional sex. Interventions should also tackle the structural constraints AGYW face so they can access resources, become empowered and challenge the expectation of having to depend financially on men. Interventions with men should address social norms, specifically those supporting the notion of men being naturally hypersexual.

## Background

Evidence points to higher incidence of HIV among adolescent girls and young women (AGYW) relative to their male peers in sub-Saharan Africa (SSA) [1]. This difference has been attributed in part to age-disparate sex [2, 3] (also referred to as intergenerational sex or cross-generational sex), [2, 4] which the literature generally defines as heterosexual intercourse with a non-marital partner ten or more years older [1, 2, 4–6], and in part to transactional sex [7]. Intergenerational relationships are primarily characterised by relationships between younger women and older men [4, 5]. The imbalance of power occasioned by age and gender, the economic realities that frequently drive such relationships and the transactional element in most of these relationships [8, 9] have also led some scholars to label sex involving older men as sexual exploitation of children, when young women (under 18) are involved [10].

From a health perspective, research evidence from SSA supports the existence of a link between age-disparate sex and HIV infection among adolescent females [6, 11, 12]. Condom use has been reported to be low in sexual relationships that involve AGYW and older men, highlighting AGYW’s constrained agency to negotiate condom use owing to unequal power dynamics within the relationships; furthermore, there is a higher likelihood that older men in these relationships are already living with HIV [6, 11, 12].

As young people transition to adulthood they begin exercising increased autonomy, including in their sexual lives, and they may decide to have sex with partners of different ages. Despite the general view that girls are disadvantaged in age-disparate sexual relationships, little has been done to understand the motivations of those who engage in them, the views of those who socialise AGYW into accepting such relationships, the perceived power dynamics and the awareness of risks inherent in these relationships. AGYW may be motivated to engage in age-disparate sex for several perceived and real benefits and may not always interpret such relationships as “exploitative”. Moreover, uni-dimensional portrayals of young women’s relationships with older men may not be adequate, and can have negative implications for intervention approaches [8].

Although interventions have targeted vulnerabilities that lead AGYW into age-disparate relationships [13, 14], not much has been achieved to stop the practice [15], nor to stop new HIV infections [16]. It is apparent that, if interventions are to respond adequately to the SRH health needs of AGYW engaged in age-disparate sexual relationships, there will be a need to thoroughly understand why AGYW and older male partners are motivated to be in such relationships.

### Theoretical framework

The social exchange theory posits that social behaviour is the result of an exchange process and that the purpose of this exchange is to maximise benefits and minimise costs [17]. The social exchange framework is useful for understanding sexuality within a relational context as it focuses on what each partner gives to and receives from the other. This framework therefore offers a way of explaining how both AGYW and men view age-disparate relationships. It assumes that both parties involved are giving and receiving items of value from each other and that the relationship will continue only if there is a positive amount of profit for both parties involved [18, 19]. Costs within sexual relationships involve things that the individual may perceive as negatives, such as having to invest money, time and effort into a relationship, whereas benefits are the things the individual gets out of the relationship, such as sexual gratification, friendship, companionship, status and social and material support [19]. Positive relationships are those in which the benefits outweigh the costs; negative relationships are those in which the costs are greater than the benefits [20]. People weigh the benefits of a relationship against its costs by establishing a comparison level that is often influenced by social expectations and past experiences [18]. AGYW may decide to have an older partner not because they like it but because it is the most feasible beneficial option they have at a particular moment within the limited opportunities that society provides them.

Female power in the context of globalisation and consumerism is part of the broader gendered system of sexual exchange and reciprocity [21]. Eroticism constitutes a significant resource that enables young women to engage actively in sexual relations with older partners to extract money and other material items [3, 22, 23]. As such, contrary to typical portrayals of AGYW as helpless and oppressed victims without power in these relationships, we need to recognise AGYW who engage in age-disparate sex as active social agents making conscious choices [24, 25]. We argue that age-disparate relationships have costs and benefits to both the AGYW and the older male partner, and that these relationships go beyond a “victims” and “villains” dichotomy and intricately intertwine love, gender, resource redistribution, agency and power. The relative levels of involvement, dependence and resources contribute importantly to the different patterns of interaction observed within exchange relationships. Therefore, drawing on the social exchange framework, AGYW and their older male partners may engage and disengage in different (sometimes concomitant) sexual relationships depending on their evaluation of the benefits and costs of each at a given point in time.

We employ the social exchange framework to understand the factors that motivate (i.e., the material and non-material benefits of) sexual relationships involving AGYW and older men and how these relationships are interpreted by the AGYW who engage in them, by older men who pursue them and by family members who tacitly permit or condone them. An understanding of the costs and benefits to AGYW and adult male partners who engage in age-disparate sex is critical for designing relevant interventions that address the “real” issues from the perspectives of those who experience these relationships and those who form part of the social context in which they occur.

## Methods

This study employed an ethnographic research design involving focus group discussions (FGDs) and in-depth interviews (IDIs). Fieldwork was undertaken in two sites in Mwanza region, Tanzania (one rural and one urban), and two sites in Uganda (Kampala and Masaka). The study population was young people aged 14–24 years in and out of school and community members aged 25–49 years. The IDIs and FGDs explored: motivations (benefits) for, and consequences (costs) of, age-disparate sex; norms and expectations of age-disparate sex; AGYW as targets for male sexual interest; understandings of sexual consent; and sexual exploitation.

### Sampling and data collection

The sites and participants were sampled to reflect a broad range of experiences and socio-demographic characteristics, including age, schooling status and type of residence.

In Tanzania, the study used both snowball and purposive sampling. Initial contacts for three unmarried young women and three unmarried young men, both in school and out of school, were the basis for a snowball sample of young people. In addition, the study sampled adult men and women (aged 25–49) through snowball and purposive sampling. In Uganda, participants were purposively sampled from the beneficiaries of a local non-governmental organisation (NGO) – the Uganda Youth Development Link (UYDEL) (www.uydel.org) – or through the local government structure.

As Table 1 indicates, a total of 37 FGDs and 87 IDIs were conducted, using semi-structured topic guides with a set of predefined questions and probes to follow up on issues as they emerged. The FGDs were conducted first. During these, questions were limited to general issues; no personal experiences were discussed. In Tanzania, a sub-sample of 23 young people and 20 adults (Table 1) were then purposively selected from the FGD participants based on willingness to participate in an IDI, their enthusiastic and low participation in the FGD and their knowledge of the discussion topics during the FGD sessions. The IDIs benefited from the rapport built during FGDs and were therefore more focused on personal experiences of age-disparate sexual relationships. In Uganda, individuals were purposively sampled from beneficiaries of UYDEL or from the local community, with a few sampled from the FGDs.

**Table 1:** Overview of Focus Group Discussions and In-depth Interviews

	Tanzania				Uganda			
	Focus Group Discussions (n = 18)		In-depth Interviews* (*n* = 43)		Focus Group Discussions (*n* = 18)		In-depth Interviews* (*n* = 43)	
Participant Category	Rural	Urban	Rural	Urban	Rural	Urban	Rural	Urban
Young women ages 14–24*								
In-school	1	1	4	3	1	1	2	2
Out-of-school	2	2	4	4	3	3	4	6
Young men ages 14–24*								
In-school	1	1	2	2	1	1	2	2
Out-of-school	1	1	2	2	2	2	4	4
Adult women(25–49 years)*	2	2	6	6	1	1	4	4
Adult men (25–49 years)*	2	2	4	4	2	1	6	4
Total	9	9	22	21	10	9	22	22

The majority of FGD participants were sampled from the community or through UYDEL

*All participants who participated in an IDI previously participated in a FGD

Given the sensitivity of this topic and cultural tradition in the study contexts, participants were interviewed by an interviewer of the same sex. IDIs and FGDs were conducted in Swahili in Tanzania and in Luganda in Uganda in a private place, and were audio recorded. The facilitators and interviewers were trained social scientists with experience collecting data on sensitive topics, including sexual behaviour, and working with vulnerable populations, including children. Ethical clearance for the study was obtained from the Medical Research Coordinating Committee of the National Institute for Medical Research in Tanzania, the Ethics Committee of the Uganda Virus Research Institute, the Uganda National Council for Science and Technology and the Ethics Committee at the London School of Hygiene and Tropical Medicine. Verbal and written informed consent was sought from all participants above 18 years. For those below 18 years, assent was sought from the young person in addition to parental consent. In Uganda, UYDEL provided in loco parentis consent for the young people under its care.

### Data analysis

The data were transcribed verbatim, translated into English, entered into QSR NVIVO 10 software and coded by three researchers involved in the data collection. A pragmatic approach to analysis was adopted, whereby combined use of a predefined coding scheme (anticipated codes) and grounded codes was utilised. The anticipated codes were developed from the research objectives, prior knowledge and repeated reading of the data during the early stages of the analysis. They were refined in light of further data. Grounded codes were developed by means of a thorough reading of the data by two researchers in consultation with the data collection team, and these codes reflected the participants’ language and ways of expressing ideas portrayed. Thereafter, codes were developed into more conceptual categories and, finally, themes [26]. Widespread views supporting the emerging theories were examined alongside the deviant cases. In the presentation of results, deviant cases are also presented as appropriate. Representative quotations illustrating the main findings were identified from a varied array of participants.

## Results

### Socio-demographic characteristics

Young people who participated in the study were aged 14–24 years. In Tanzania, all of the young people were unmarried and resided with either biological parents or other relatives. In Uganda, out-of-school young people aged 14–17 were in the care of UYDEL, and all young people were unmarried. Most AGYW from the rural setting were engaged in farming and petty trade, whereas those from the urban setting were engaged in petty trade or did not have specific activities that earned them an income. Adult participants were aged 25–49 years and the majority were married. The rural male participants were peasant farmers sometimes engaged in fishing and petty trade, whereas those from the urban setting were petty traders or involved in informal employment (e.g. motorbike transport business).

### Reflections on the perceived benefits and costs of AGYW engaging in age-disparate sex

Content arising from the IDIs and FGDs related to AGYW’s motivations, perceived benefits and costs with regard to engagement in relationships with older men was organised around four main themes: financial motivations, emotional support, meeting social expectations and, finally, AGYW’s reflections on sexual health.

#### Financial motivations

Across all settings, AGYW described being motivated to engage in relationships with older men for two primary reasons. First, older men were thought to provide better and more reliable resources as compared with younger men. This was particularly the case in urban Uganda, where the high cost of living and, for some, limited social capital resources meant people struggled to meet their needs. Second, AGYW noted that older partners could be trusted to honour their promises of gifts and gave bigger gifts and larger amounts of money compared with same-age boyfriends. AGYW perceived their relationships with older men as an important means by which they could meet their needs as well as maintain their social position within their peer networks.

*No, the reason why we do not take on men of our age is that these boys who are of our own age are so mean. When you have sex with him, he only gives you 5000/= [$1.33] at the end yet you admired a certain dress that your friend Dora has. Dora’s dress does not cost 5000/=... that is why you get a daddy who will tell you that let us go to the shop and you point at the dresses then they pack them for you. When you go to the shop you even ask for the shoe that Prossy has. [FGD, young women aged 18-24, urban Uganda]*

#### Emotional support

AGYW interpreted the provision of gifts or money as synonymous with “care” and emotional support, particularly where they experienced stress and anxiety in trying to meet their needs. Many AGYW emphasised that older men cared for them more than same-age peers did:

*R1: You find that the older one cares for you… you just decide, “I would rather be with this one who cares for me… He gives me what I want.”*

*R2: I think that also contributes because you can’t have a relationship with a boy who is just there, and all he wants from you is sex and does not care [provide material needs] for you… You can’t continue being with him. [FGD, adolescent girls aged 14-17, urban Tanzania]*

In Uganda, younger women expressed love from older partners as follows:

*Girls choose to be in relationships with men who are older because those older men have too much love yet these younger boys jump around with any girl. If he sees a girl who is his age, he wants to initiate a relationship with her as well. The older man has love, the girl knows that he will always be close by, he is responsible enough to buy the things that you need at home because he feels that you are young, if he neglects you, other men will take you away. [FGD, young women aged 18-24, urban Uganda]*

AGYW and adult men felt that older partners were more likely than younger men to keep their promise to marry their partners.

*There is a difference between the one who is an age mate same age and the one who is a much older one… The younger one plays with you and dumps you… but the older man shows true love and may even marry you. [FGD, adolescent girls aged 14-17, urban Tanzania]*

#### Sexual and reproductive health

The majority of participants interviewed from all categories agreed that AGYW in age-disparate sexual relationships faced many sexual and reproductive health (SRH) risks. Overall, they felt there were more costs than benefits to girls in age-disparate sexual relationships. They talked about adult men being infected with HIV and other sexually transmitted infections (STIs) and as being the ones likely to infect AGYW. Recognising these risks, adult men reported:

*I think is it mainly due to lack of knowledge… There is no benefit that they get in relationships with older men, it is mostly problems… She loves him because of his money, now you find that this man has already been infected for a long time… She gets those viruses… She can also become pregnant… thinking about the amount of Tsh 50,000 [$23], but it is not even enough to treat her of STIs, even though she may think that it is a big benefit, but it’s actually a loss. [FGD, adult men, urban Tanzania]*

However, such awareness does not prevent AGYW from having relationships with older men. An 18-year-old woman talked about this in the following way:

*Being with that older man has consequences but he thinks about the money he gives you and feels that he is justified to manipulate you… He knows that you can’t refuse when he tells you to have sex… He manipulates you the way he wants and at any time that he may desire. [IDI, young woman aged 18, rural Tanzania]*

Overall, men are the decision-makers in sexual relationships and AGYW were aware of this. Many AGYW talked of having unprotected sex because they did not have the power to enforce the use of protection. A 17-year-old girl reported:

*It is the men who decide on that [protection use] because you can’t force him to use protection yet he is the one who buys protection, what if he refuses and says he doesn’t have money to buy it, what will you do? [IDI, adolescent girl aged 17, urban Tanzania]*

While many AGYW described having limited agency in relationships with older men, a few noted that an unplanned pregnancy with an older man would likely be better than one with a younger man, as one girl from rural Uganda explained:

*Most of the girls are interested in men who are working, so that in case he makes the girl pregnant or any other problem and her parents ask her to bring the man that made her pregnant so that he can marry you... If it was a fellow student who is in senior three or senior four [secondary school], he will deny the pregnancy and he will abandon her... Yet if it was the older man he will stick with her, that is if he truly loved her, he can take her and marry her... You are secure with the older man. [FGD, in-school girls, rural Uganda]*

While being older does not necessarily guarantee that a man will provide for them, AGYW perceived older men as having access to more resources and thus support to deal with any challenges that an AGYW might experience.

#### Meeting social expectations

Peer pressure seems to play a role in AGYW’s partner choices, and comes with social costs. Those whose boyfriends are peers are sometimes ridiculed for not being strategic in terms of selecting a partner who has resources. In describing how older partners ensured better access to resources, school girls from rural Uganda reported:

*Some girls say that I cannot enter into a relationship with mulinya decker [one who sleeps on a decker/bunk bed] or their age mates, the ones that we are at school with, because they know that he will have to ask for money from his parents, yet the girl is also asking for the money from her parents... so what will he be able to provide her with? They want the other men who have already earned their money, one whom you will ask to give you 50,000/= [$13.30] and he will not hesitate even for a minute. [FGD, in-school girls, rural Uganda]*

The in-school girls further described the embarrassment of being with an age mate:

*Some girls do not want to be in a relationship with their age mates because they might be in the same class, then they [teachers] ask him to lie down and they cane him, so the girl feels “shamed” [embarrassed], even the other students who do not have boyfriends can start rebuking her, so she gets hurt. Even the others who get to know about it also say that they cannot have a relationship with a student after realising what this one is going through. [FGD, in-school girls, rural Uganda]*

While some AGYW described being strategic in their choice of an older man, others valued relationships with their age mates, which were more often described, particularly by those still in school, as emotionally intimate or based on a shared life stage, for example aspirations to finish school and then get married.

Beyond this, however, AGYW pointed to the social expectation that the provision of gifts and money in relationships had to be reciprocated, hence the expression: “*Eat and you will be eaten*”. “Eat” refers to receiving gifts and money from men whereas “you will be eaten” means men having sex with the girls. Young women reported:

*“Eat and you will be eaten”… And you have to pay back by labouring through having sex with him whether you like it or not… It means, “Help me and I help you.” [FGD, young women aged 18–24, rural Tanzania]*

In both countries, this expectation of reciprocity means that, if an AGYW receives gifts or money, this is perceived as implicit agreement of her obligation to provide sex. In situations where a girl is not interested in having sex with the older man after receiving material benefits, the man will appeal to the social expectation of reciprocity by reminding the girl of the gifts and money and ultimately using this as a tool of coercion. A young woman recounted:

*He forces you… You might find that those whom they have sex with are older than them and give them things [money/gifts]. They will start to remind you, “I always give you this and that, how comes then today you have refused to have sex with me?”, you will have to do it... It is not fair… She agrees to do it but in her heart she is not actually willing to have sex. [IDI, adolescent girl aged 17, urban Tanzania]*

In Uganda, AGYW who deliberately avoid fulfilling this expectation were said to have ‘de-toothed’ a man, which was also described as putting them at risk of sexual violence.

*[By not providing sex] she is also still taking a risk. You might proudly say that I ate his things [accepted his money] and did not pay him [with sex], but then he finds you along the way and reminds you of his things and, even if you try to escape, there is no way out, he may not be alone… and they rape you. He can even end up infecting you with HIV yet you refused to do it [give him the sex you were obligated to give] peacefully. [IDI, in-school young woman aged 18, urban Uganda]*

As an additional social cost, all participants in Tanzania, including AGYW, reported that having sex with older men tarnished girls’ reputation in the community and led to stigmatising labels such as *malaya* (prostitute). AGYW in such relationships are compelled to handle their engagements discretely. A 20-year-old woman in a sexual relationship with a 40-year-old man talked about guarding her reputation:

*I have never told anyone I am having sex with an older man… It is embarrassing to tell people… I am not compatible with him because my body is small and he is big… I just keep it to myself. [IDI, young woman aged 20, rural Tanzania]*

Some AGYW described such relationships as bad because of the shame and embarrassment of being seen with a much older man. One reflected on the social costs arising from the age gap in the following way:

*The reason why I say that it is bad is because that man could be in love with you seriously then he asks you to take him to your home and when you get there, your husband is even older than your father; it is embarrassing. [FGD, young women, aged 18-24, urban Uganda]*

### Reflections on the perceived benefits and costs for older men engaging in age-disparate sex

Men’s perceived benefits and costs resulting from engaging in relationships with younger women revolved around the need to satisfy their sexual desire; the social expectations associated with having a young sexual partner; and sexual health beliefs.

#### Need to satisfy sexual desire (*tamaa*)

Men talked about experiencing increased desire for sex (*tamaa*) as they aged. When describing older men’s desire to have sex with younger women as opposed to their own wives, who were perceived as old, adult men reported:

*You might pass through other stages of life quietly, but when you reach old age, you start experiencing temptations; these desires might even double up those you experienced when you were young. You might get money during old age and that increases your desire to get whoever passes by, you see! But during childhood you never experienced such desires... but as you age, you find that you are “out of control”. [FGD, adult men, urban Tanzania]*

Adult women agreed with the views of older men. Reflecting on their own relationships, married women observed that their husbands considered AGYW more desirable than them. The reasons for considering AGYW desirable were: the perception that they had “tight” vaginas; AGYW offered more pleasurable sex than adult women, and were cleaner and neater than married women; and AGYW were attractive, presentable and pleasant to spend time with:

*An older man will handle her well because he wants that small vagina that holds him tight [laughter] and he also feels good because of that, that’s what the man wants. [FGD, young men, aged 15-24, rural Uganda]*

Adult women mentioned that most of them were busy fending for their families and were involved in manual work; thus, they were often tired and not able to be as active during sex as their husbands preferred:

*For example, an old woman like me, I am not soft/smooth [attractive], I am tired from doing farm work… I have been farming for the whole day and I tell him, “Today I am very tired”… but now for a little girl, she can handle and accepts it every day as long as the man wants it [sex]. [FGD, adult women, rural Tanzania]*

#### Societal tolerance for men’s behaviour

It is socially accepted for older men to date or even marry younger women. Some adult men reported that love did not discriminate and therefore the age of a girl did not hinder them from having a sexual relationship with her. Adult women described the double standards that existed in their communities with regard to social expectations that allow older men to have younger partners but disapprove of older women partnering with younger men. They described the prestige deriving from adult men having sexual relationships with AGYW:

*In our community, many men do not see this as bad… They see it as just normal, again they boast/praise themselves… “I have a young/little one, chap!”… but now, when an older woman does this, it becomes a big issue that must be heard in newspapers [gossip]. [FGD, adult women, urban Tanzania]*

Older men reflecting on the costs of engaging in relationships with younger women blamed themselves for having been involved with young women. The majority of adult men admitted that the short-term benefits of pursuing younger women (satisfying sexual desires) were outweighed by the long-term costs to their reputation. Describing the stigma facing older men who engage in sexual relationship with younger women, men in Tanzania further described such relationships as *uchafuzi wa mazingira*, a term that can be translated roughly as “polluting the environment”. Such relationships also lead to shaming of the man involved:

*There are losses… The number one loss of being in those relationships is the embarrassment due to age differences… You also lose respect… Loss number two is when the girl is made pregnant by that older man, and she mentions a man whom people know has a family, has older children and grandchildren. [FGD, adult men, rural Tanzania]*

#### Economic, psychological and sexual health

While men talked about the pleasure they obtained from having sexual relationships with younger women, they also reflected on the economic and psychological costs of these relationships. One man said:

*Personally, the benefits for me being in a relationship with her were those short-lived pleasures during the time I was with her. But after leaving her I always regret about the money I gave her… You see I use my money for a short time and later I am really worried that probably my wife will know about the affair but also what if the community knows about this? You see, I end up just blaming myself. [IDI, adult man, involved in age-disparate sex, aged 35, rural Tanzania]*

Weighing the perceived benefits (disease-free) versus the real costs (AGYW having infections) of having sex with young women, adult men reported:

*Another problem is that for us men, we believe that those of a young age are safe and hence we have sex without a condom... because we think this one has no infections she is young… Unfortunately, he [adult partner] gets those infections from her [FGD, adult men, rural Tanzania]*

Considering the health costs of engaging in age-disparate relationships, adult men expressed awareness of risks such as unplanned pregnancy and STIs including HIV. They talked about a larger proportion of AGYW being infected compared with other age groups and said that they were the ones passing on the infections to adult men.

*Many more little girls have already been infected with HIV than adults… Now, because of his money, he [an adult man] decides to be with that girl without knowing she has the viruses… By doing that he is finished [infected]. [FGD, adult men, rural Tanzania]*

## Discussion

We set out to explore the motivations of AGYW and older men engaging in age-disparate sexual relationships and their perceptions of the social and SRH costs of these relationships. Our results show that age-disparate sex is common, tolerated privately but condemned at the public level and may be considered by young women as more attractive and beneficial than sexual relationships with men in their age group. Our findings also indicate that AGYW and older men are motivated to be in these relationships for multiple reasons, with each group expressing some degree of control in the relationship. AGYW’s central motivations for engaging in sexual relationships with older men are material benefits and the better treatment older men offer AGYW partners. As observed in this study, older men are usually the ones with resources, and provide for AGYW. As noted in other studies, those with more resources also tend to be the ones with the greatest decision-making power [4, 27, 28].

AGYW perceived older partners as readily and generously providing when called upon compared with younger men. There seems to be little trust in these relationships. Trust is important in relationship development because it allows individuals to be less calculative and to realise long-term outcomes in the relationship [29, 30]. It has been noted that, when relationships conform to the norms of reciprocity and when the pattern of exchange is perceived as fair, individuals are more likely to trust their partners, as they believe they will not be exploited in a relationship [29].

Unlike young women, older men engage in age-disparate sex for prestige and pleasure and out of *tamaa* (sexual desire). Adult women and men also described marital challenges like wives being too tired to actively participate in sex, with husbands as a result looking for younger women. Therefore, male *tamaa,* the desire for prestige and the perception that AGYW offer more pleasurable sex compared with older women motivate older men to engage in sex with AGYW. The discussion of *tamaa* as a key driver of sexual decision-making, especially among AGYW engaging in sexual relationships with older partners, has previously been observed in Tanzania [21, 31].

Reciprocity is expected in sexual relationships, and AGYW’s acceptance of gifts and/or money from their partner implies readiness for sex with him when he wants it. Participants used the expression “*Eat and you will be eaten*” to explain the social expectations around reciprocity, to describe the risk associated with not honouring these reciprocal expectations. Receiving gifts and money from men makes AGYW very vulnerable and jeopardises their ability to make decisions in the relationship, thus limiting the initial agency they exercised in their choice of partner.

Although some sexual relationships involve aspects that disadvantage AGYW, most of the encounters reported were consensual. Therefore, although sexual relationships involving younger women and older male partners are condemned, it is apparent that younger women may be motivated to engage in these relationships for a variety of benefits.

An interesting finding revolves around the study participants’ perceptions of the social and SRH costs inherent in age-disparate sexual relationships. The most common social costs mentioned were the stigmatisation of AGYW and older men who engage in these relationships. Although such behaviour is condemned at the public level, it is tolerated at the individual level as well as within peer groups. The stigmatisation of age-disparate sexual relationships does not prevent older men and AGYW from engaging in them but rather results in secrecy. These findings are similar to what has been observed in other studies on contradictory sexual norms [21, 22, 32], and represent a barrier to the prevention of harmful sexual practices.

Participants articulated the SRH health costs for AGYW and older men engaging in age-disparate sexual relationships. The most commonly discussed SRH risk was HIV, with each group blaming the other as being likely to be infected and hence likely to infect them. Therefore, while most AGYW and community participants perceived older men involved in age-disparate sexual relationships as taking advantage of AGYW, some older men had different views. They described AGYW as a source of HIV infection for them. Even though there is an awareness of the risks inherent in age-disparate sexual relationships, the “blame game” and power differences are observed here, and have also been observed in other studies [4, 11] – and may have resulted in a lack of meaningful communication about the use of condoms.

These findings provide insights on the motivations of and consequences for AGYW and older men engaging in age-disparate sexual relationships, based on lived experience as well as from the point of view of others in the community. Community views are important since they sustain norms in support of, as well as against, these relationships.

This study has strengths and limitations. Sampling from both rural and urban areas in Tanzania and Uganda provided an opportunity to explore the study themes with a variety of study participants in a range of settings. The similarities in the data from these two countries suggest that the themes explored may be salient in other countries and contexts with similar socio-economic and cultural profiles. The sample sizes were small, however, which limits comparison across socio-demographic groups. Repeated interviewing with participants would likely have also provided an opportunity for researchers to build more rapport with participants, which may have been valuable given the sensitivity of the themes explored.

## Conclusion

Despite awareness of the negative health and social consequences of age-disparate sex, AGYW and older men are motivated to engage in these relationships for several reasons. There is a need for interventions to acknowledge the perceived benefits of age-disparate sex from the perspective of the AGYW and older men who engage in it. For girls, the financial and emotional benefits do not seem to present direct associated costs; for men, the sexual desire benefits do not seem to present any direct associated costs. Given the restrained access to material goods for girls and the important role hypersexuality plays in the construction of masculinities, it seems clear that transactional sex offers benefits in areas that are particularly salient for girls and men. Hence, interventions need to focus on both AGYW and older male partners, to build skills in critical reflection on the short-, medium- and longer-term benefits and costs of engaging in age-disparate sexual relationships.

The economic aspect is key to adolescent girls’ engagement in age-disparate relationships, although some relationships are motivated by emotion. Interventions such as cash transfers could address the economic aspect for the most vulnerable girls. However, we still need to better understand the impact of cash transfers [33], and there is a need to combine cash transfers with gender-transformative sessions, financial literacy training and female entrepreneur role modelling to provide girls with a concrete example of how they can access resources in alternative ways.

In terms of interventions with men, working on the issue of hypersexuality as defining manhood, as well as men’s understanding of girls’ readiness for sex not only in bodily terms but also in emotional and cognitive terms, is important.

Finally, recent evidence from the Global Early Adolescent Study (GEAS) shows that these results could guide curriculum development for a critical reflection process drawing on what people perceive as beneficial and working with what they value in age-disparate relationships, to help them adopt safer sex behaviour [34]. This would guide them in reflection on the short- and long-term consequences of their decisions, especially in age-disparate relationships.

Since the motivations for AGYW and older men’s engagement in age-disparate relationships are multi-dimensional, interventions targeting this practice should be multi-component, engaging in different ways with various groups to address the role they play in perpetuating these relationships, or to bolster the role they could play in preventing them. Specifically, SRH interventions and broader social development programmes could utilise these findings to help AGYW explore pathways into their future and assist them to understand how age-disparate transactional sex could interfere with those plans, even if it seems to facilitate access to more immediate goals. AGYW have some degree of agency in their sexual relationships, and interventions should tap into this to encourage negotiation and use of condoms, while at the same time making condoms and pre-exposure prophylaxis available to AGYW. These results also point to the need for community-based SRH and gender programmes to consider social norms around gender and power that influence age-disparate relationships.

## Abbreviations

AGYW: Adolescent Girls and Young Women
FGD: Focus Group Discussion
GEAS: Global Early Adolescent Study
HIV: Human Immunodeficiency Virus
IDI: In-Depth Interview
NGO: Non-Governmental Organisation
SRH: Sexual and Reproductive Health
SSA: Sub-Saharan Africa
STI: Sexually Transmitted Infection
UYDEL: Uganda Youth Development Link
